# Microwave–vacuum drying modulates the supramolecular organization and biofunctionality of β-glucans from edible mushrooms

**DOI:** 10.1016/j.fochx.2026.104285

**Published:** 2026-08-04

**Authors:** Ociel Muñoz-Fariña, Luis Ríos, Patricio Arce-Johnson, Luisbel González, María Cristina Ravanal

**Affiliations:** a Instituto De Ciencia y Tecnología De los Alimentos, Facultad De Ciencias Agrarias y Alimentarias, Universidad Austral De Chile, Valdivia 5090000, Chile; b Escuela De Graduados, Facultad De Ciencias Agrarias y Alimentarias, Universidad Austral De Chile, Valdivia 5090000, Chile; c Instituto De Ciencias Aplicadas, Facultad De Ingeniería, Universidad Autónoma De Chile, Santiago 8581151, Chile; d Facultad De Agronomía y Sistemas Naturales, Pontificia Universidad Católica De Chile, Santiago 7820436, Chile; e Instituto para el Desarrollo Sustentable, Pontificia Universidad Católica de Chile, Santiago 7820436, Chile

**Keywords:** Microwave–vacuum drying, Hot-air drying, Mushrooms, β-glucans, Supramolecular organization, Antiproliferative activity

## Abstract

Microwave–vacuum drying (MVD) was evaluated as a structuring strategy for β-glucans from *Agaricus bisporus*, *Cyttaria espinosae*, and *Grifola gargal*, using hot-air drying (HAD) as reference. MVD markedly intensified dehydration, increasing Page model drying constants by up to 20–40-fold compared with HAD, with excellent fitting (R^2^ = 0.9980–0.9995). Fresh β-glucan contents were species-dependent, reaching 55.02 ± 3.61 g/100 g d.b. in *G. gargal*, 31.00 ± 1.13 g/100 g d.b. in *C. espinosae*, and 9.42 ± 1.40 g/100 g d.b. in *A. bisporus*. FTIR confirmed preservation of the β-glucan chemical fingerprint, whereas DSC and SEM revealed drying-dependent supramolecular reorganization. All extracts were cytocompatible toward HDF cells. Notably, MVD-derived *C. espinosae* β-glucans reduced AGS and Caco-2 viability to 31.03 ± 4.90% and 41.27 ± 7.60%, respectively. These findings support MVD as a promising strategy to tailor mushroom β-glucan biofunctionality.

## 1 Introduction

Edible mushrooms are increasingly recognized as more than culinary commodities, serving as sources of structurally diverse polysaccharides with biofunctional potential. Among these, fungal β-glucans—typically enriched in β-(1 → 3) backbones with β-(1 → 6) branching—have attracted sustained attention due to their immunomodulatory and anticancer-associated activities, which can include both direct effects on tumor cell behavior and indirect effects mediated through innate immune recognition and downstream cytokine signaling (Noorbakhsh Varnosfaderani et al., 2024). The biological performance of β-glucans is not dictated solely by “β-glucan content”, but strongly depends on higher-order features such as chain conformation, aggregation state, hydration/packing, and the accessibility of bioactive motifs at interfaces—properties that ultimately control how β-glucans engage receptors and cellular pathways (Venturella et al., 2021). In this context, Dectin-1 is a major β-glucan receptor implicated in fungal-pattern recognition and immunological signaling, providing a mechanistic basis for why structural presentation can translate into markedly different functional outcomes (Brown, 2006).

Chile hosts a distinctive mycobiota that includes culturally and nutritionally relevant wild edible species such as *Cyttaria espinosae* (“digüeñe”) and the native polypore *Grifola gargal* (“gargal”), which represent underexploited resources for value-added bioproducts (Vetter, 2023). (However, like most high-moisture fungi, these matrices are highly perishable and seasonal, making stabilization a prerequisite for broader utilization and for building consistent supply chains beyond local and short harvest windows (Ortiz, 2013). Drying is among the most practical preservation strategies, yet it is also a strong “structuring driver” that can reshape microstructure, browning chemistry, and extractability of carbohydrate fractions—changes that may propagate into the organization and functionality of extracted β-glucans.

Conventional hot-air drying (HAD) is widely used but often entails long exposure times and can promote shrinkage/collapse, thermal degradation, and color deterioration, particularly in complex, protein- and sugar-containing matrices where Maillard-type pathways are kinetically favored as water activity evolves. In contrast, microwave–vacuum drying (MVD) couples volumetric dielectric heating with reduced-pressure evaporation, accelerating dehydration and frequently improving structural retention relative to convective approaches, while operating at lower apparent boiling temperatures under vacuum (Giri & Prasad, 2007). For mushrooms specifically, MVD has been repeatedly shown to intensify drying kinetics and to influence quality attributes and microstructural outcomes compared with hot-air methods, supporting its relevance as an industrially meaningful alternative.

Despite this, a key translational gap remains: drying is typically evaluated in terms of time–energy efficiency and visible quality, whereas the process–structure–function consequences for mushroom β-glucans are far less systematically addressed—particularly for native Chilean species. Emerging work on *C. espinosae* confirms that its polysaccharides are bioactive and measurable across different endpoints, but the extent to which drying-driven physical reorganization (rather than backbone chemistry) modulates β-glucan supramolecular state and downstream biological potential remains underexplored (Pérez et al., 2025). In oncology-oriented applications, this question is especially relevant: β-glucans are increasingly framed as bioactive ingredients and adjuvant-type agents in digestive-cancer contexts, yet their performance is tightly coupled to accessibility and assembly state—features that processing can plausibly tune without changing the chemical “fingerprint” (Wang et al., 2024).

Accordingly, this study hypothesized that the drying route can modulate the supramolecular organization and biofunctionality of mushroom β-glucans without altering their characteristic chemical fingerprint. To test this hypothesis, the drying kinetics of *Agaricus bisporus*, *Cyttaria espinosae*, and *Grifola gargal* processed by hot-air drying (HAD) and microwave–vacuum drying (MVD) were compared using Page-model parameters. The effects of the drying conditions on instrumental color, free sugars, and glucan composition were also determined, while drying-induced changes in the thermal behavior, microstructure, elemental composition, and spectroscopic fingerprint of the extracted β-glucans were characterized using DSC, SEM/EDS, and FTIR. Finally, selected *C. espinosae* β-glucan extracts were evaluated for cytocompatibility in human dermal fibroblasts and for antiproliferative activity in AGS and Caco-2 gastrointestinal cancer cells. Through this integrated approach, the study aimed to establish relationships among drying conditions, β-glucan supramolecular organization, and cellular responses.

## 2 Materials and methods

### 2.1 Materials and reference β-glucans

Commercial β-glucans were used as analytical and biological reference materials. These included β-1,3-glucan from *Euglena gracilis* (Sigma-Aldrich; product code 89862) and β-D-glucan from barley (Sigma-Aldrich; product code G6513). The molecular-weight values associated with these reference materials correspond to manufacturer-reported information (500 and 376 kDa, respectively).

For glucan quantification, the β-Glucan Assay Kit (Yeast and Mushroom) (K-YBGL; Megazyme, Bray, Ireland) was used according to the manufacturer's protocol. This assay is designed for the indirect determination of 1,3:1,6-β-glucan in mushroom, yeast, and algae preparations and includes a yeast glucan control for routine assay verification. Additional reagents used for extraction, clarification, purification, and analysis included ethanol (CAS 64–17-5), n-hexane (CAS 110–54-3), chloroform (CAS 67–66-3), and n-butanol (CAS 71–36-3). For sugar clarification, Carrez I reagent was prepared from potassium hexacyanoferrate(II) trihydrate (CAS 14459–95-1) and Carrez II reagent from zinc sulfate heptahydrate (CAS 7446-20-0). Sugar quantification was performed using analytical standards of arabinose, fructose, and glucose.

Fresh fruiting bodies of *Agaricus bisporus*, *Cyttaria espinosae*, and *Grifola gargal* were obtained from local markets in Valdivia, Chile. Upon arrival, samples were manually cleaned to remove adhering debris and damaged tissue, vacuum-sealed in food-grade bags, and stored at −80 °C until further processing.

### 2.2 Sample preparation and initial moisture content

Prior to drying, mushrooms were equilibrated to room temperature and sliced to a uniform thickness of 5 mm using an electric slicer to minimize geometric variability. Initial moisture content was determined gravimetrically according to AOAC methodology by oven drying at 105 °C to constant mass. All measurements were performed in triplicate, and results were expressed on a dry basis.

### 2.3 Drying procedures: hot-air drying (HAD) and microwave–vacuum drying (MVD)

Drying experiments were performed using hot-air drying (HAD) and microwave–vacuum drying (MVD). HAD was conducted in a convective laboratory oven (Biobase, BOV-V65C) at air temperatures of 40, 60, and 80 °C, with samples arranged in a single layer on perforated trays to ensure uniform airflow. MVD was carried out in a microwave–vacuum dryer (ZZKD, Zhengzhou Keda, China) at microwave powers of 250, 350, and 450 W under reduced pressure (16 kPa), covering low-, medium-, and high-intensity regimes while avoiding visible scorching. For both methods, mass loss was monitored gravimetrically using an analytical balance (A&D GF-400, ±0.001 g); measurements were taken at progressively increasing intervals during HAD (15–60 min, depending on drying stage) and every 2 min during MVD due to faster kinetics. Drying was continued until constant weight was reached, and all experiments were conducted in triplicate. Dried samples were immediately cooled in a desiccator, vacuum-packed, and stored at room temperature in the dark until further analyses.

### 2.4 Drying kinetics and thin-layer modeling

Drying kinetics were described in terms of moisture ratio (M_R_), calculated as M_R_ = (M_t_ − M_e_)/(M_0_ − M_e_), where Mt. is the moisture content at time t, M_0_ is the initial moisture content, and Me is the equilibrium moisture content. Given the relatively low Me values under the experimental conditions, M_R_ was simplified to M_R_ = M_t_/M_0_. Experimental M_R_ data were fitted using the Page model, M_R_ = exp.(−k·tⁿ), where k is the drying rate constant (min⁻^1^), n is the empirical model exponent, and t is drying time (min). Model parameters were estimated by nonlinear regression, and goodness of fit was evaluated using the coefficient of determination (R^2^), root mean square error (RMSE), and reduced chi-square (χ^2^). The Page model was selected based on its consistently superior performance across drying conditions and species. All kinetic analyses were performed using triplicate datasets, and fitted parameters are reported as mean values.

### 2.5 Instrumental color (CIELAB)

Instrumental color was determined on fresh and dried mushroom samples using a colorimeter (HunterLab ColorFlex, Hunter Associates Laboratory, USA) operating with D65 illuminant and a 10° standard observer. Prior to measurements, the instrument was calibrated with a standard white reference tile. Samples were ground to a fine powder to minimize surface heterogeneity, and color coordinates L* (lightness), a* (red–green), and b* (yellow–blue) were recorded in triplicate at different positions. Chroma (C*), hue angle (h°), total color difference (ΔE), and browning index (BI) were calculated from L*, a*, and b* values using standard equations. Results are reported as mean ± standard deviation.

### 2.6 Free sugar extraction and quantification

Free sugars were extracted from fresh and dried mushroom samples following an aqueous extraction–clarification procedure. Briefly, 1 g of dried material or 5 g of fresh tissue were mixed with distilled water and incubated at 60 °C for 30 min under continuous agitation. The extracts were clarified by sequential addition of Carrez I and Carrez II reagents, diluted to a final volume of 50 mL with distilled water, and filtered prior to analysis. Individual sugars (arabinose, fructose, and glucose) were quantified by high-performance liquid chromatography equipped with an evaporative light-scattering detector (HPLC–ELSD). Quantification was performed using external calibration curves prepared with analytical-grade standards. Results are expressed on a dry basis as mean ± standard deviation of triplicate determinations.

### 2.7 Extraction, purification, and quantification of β-glucans

#### 2.7.1 Extraction, purification, and quantification of β-glucans

β-Glucan-enriched polysaccharide fractions were obtained from fresh and dried mushroom samples by hot-water extraction, ethanol precipitation, defatting, deproteinization, and dialysis, following previously reported procedures with modifications (Abreu et al., 2019; Pérez et al., 2025). Dried samples were extracted using a sample-to-water ratio of 1:10 (*w*/*v*), whereas fresh tissues were processed at 5:10 (w/v). The higher mass of fresh tissue was selected to compensate for its high moisture content and to provide an approximately comparable dry-matter loading in the extraction medium.

The samples were mixed with distilled water and heated at 120 °C for 40 min. After cooling to room temperature, the suspensions were centrifuged at 5000 ×*g* for 15 min at 4 °C to separate the insoluble material. The recovered supernatants were mixed with 96% ethanol at a supernatant-to-ethanol ratio of 1:1 (*v*/v) and maintained without agitation at 4 °C for 24 h, following precipitation conditions previously applied to polysaccharides from *Cyttaria espinosae* (Pérez et al., 2025). The resulting polysaccharide-rich precipitates were recovered by centrifugation at 5000 ×*g* for 15 min at 4 °C and dried at 40 °C to constant mass.

The crude polysaccharide fractions were defatted with n-hexane at a sample-to-solvent ratio of 1:2 (*w*/*v*) under orbital agitation at 150 rpm and 25 °C for 24 h. The suspensions were subsequently centrifuged at 5000 ×*g* for 10 min, the hexane phase was discarded, and the recovered solid fractions were maintained under a fume hood until complete removal of the residual solvent. The defatted fractions were then redissolved in distilled water at a concentration of 10 mg/mL.

Proteins were removed using the Sevag method, adapted from procedures previously applied to fungal polysaccharides (R. Chen et al., 2026). The Sevag reagent consisted of chloroform and n-butanol at a ratio of 4:1 (*v*/v). The aqueous polysaccharide solution was mixed with the Sevag reagent at an aqueous-phase-to-Sevag ratio of 4:1 (v/v) and agitated at 200 rpm for 30 min at 25 °C. The mixture was subsequently centrifuged at 5000 ×*g* for 5 min at 20 °C to separate the aqueous and organic phases. The upper aqueous phase was carefully recovered, while the lower organic phase and the proteinaceous material formed at the interface were discarded. This procedure was repeated three times or until no visible proteinaceous layer remained at the aqueous–organic interface.

The deproteinized aqueous fractions were transferred to dialysis membranes with a molecular-weight cutoff of 12 kDa and dialyzed against distilled water for 24 h at 4 °C under continuous agitation at 80 rpm. The dialysis procedure was based on conditions previously employed for mushroom β-glucans and fungal polysaccharides (Abreu et al., 2019; R. Chen et al., 2026). The external water volume was at least 100-fold greater than the volume of the polysaccharide solution and was replaced every 6 h, resulting in four water replacements over the dialysis period. After dialysis, the retained fractions were recovered and dried at 40 °C to constant mass to obtain the β-glucan-enriched polysaccharide fractions.

Total and α-glucan contents were quantified using the β-Glucan Assay Kit for yeast and mushrooms (K-YBGL, Megazyme, Bray, Ireland), following the manufacturer's instructions. Absorbance was measured at 510 nm using a UV–Vis spectrophotometer (Jasco V-730), and β-glucan content was calculated as the difference between total and α-glucan contents. All determinations were performed in triplicate, and the results are expressed as mean ± standard deviation.

### 2.8 Thermal, microstructural, and spectroscopic characterization of β-glucans

Thermal transitions of purified β-glucans were analyzed by differential scanning calorimetry (DSC) using a Mettler Toledo DSC822e instrument under nitrogen atmosphere. Approximately 4–6 mg of each sample were sealed in aluminum pans and heated from 20 to 250 °C/min at a rate of 10 °C/min. Onset (Tonset), peak (Tpeak), and endset (Tendset) temperatures, as well as transition enthalpy (ΔH), were obtained from thermograms using the instrument software. A cooperativity parameter (R) was calculated as the ratio between ΔH and the temperature range of the transition to assess supramolecular organization.

Microstructural features were examined by scanning electron microscopy (SEM), and elemental composition was assessed by energy-dispersive X-ray spectroscopy (EDS). Dried β-glucan powders were mounted on aluminum stubs using carbon tape and sputter-coated with a thin gold layer prior to imaging. SEM micrographs were acquired at representative magnifications, and EDS spectra were recorded from selected areas to determine elemental composition.

Fourier-transform infrared (FTIR) spectra were recorded to evaluate chemical fingerprints and conformational features of β-glucans. Measurements were performed in the 4000–400 cm⁻^1^ range using an FTIR spectrometer operating in ATR mode, with a spectral resolution of 4 cm⁻^1^ and accumulation of 32 scans per sample. Spectra were baseline-corrected and normalized prior to analysis.

### 2.9 Cell culture and viability assay

Human dermal fibroblasts (HDF) and gastrointestinal cancer-derived cell lines (AGS and Caco-2) were used to evaluate cytocompatibility and antiproliferative effects of β-glucan extracts. Cells were cultured under standard conditions (37 °C, humidified atmosphere with 5% CO_2_) in complete growth medium and subcultured routinely. For viability assays, cells were seeded in 96-well plates and allowed to adhere overnight. Cultures were then treated with reference β-glucans or *C. espinosae* β-glucan extracts at concentrations ranging from 15.625 to 1000 μg/mL.

Cell viability was determined using a Cell Counting Kit-8 (CCK-8, water-soluble tetrazolium assay), following the manufacturer's instructions. Briefly, after treatment, CCK-8 reagent was added to each well and plates were incubated to allow conversion of WST-8 into a water-soluble formazan product by metabolically active cells. Absorbance was measured at 450 nm using a microplate reader, and viability was expressed as percentage relative to untreated controls. All treatments were performed in triplicate, and results are reported as mean ± standard deviation. Cytotoxicity was interpreted according to ISO 10993-5 criteria, considering values below 70% viability as indicative of cytotoxic response.

### 2.10 Statistical analysis

All experiments were performed in triplicate, and results are expressed as mean ± standard deviation. Statistical analyses were conducted using OriginPro software. Normality and homogeneity of variances were assessed prior to hypothesis testing. Differences among treatments were evaluated by one-way analysis of variance (ANOVA), followed by Tukey's post hoc test when significant effects were detected. For datasets not meeting parametric assumptions, the Kruskal–Wallis test followed by Dunn's multiple comparison was applied. Statistical significance was defined at *p* < 0.05.

## 3 Results and discussion

### 3.1 Drying kinetics and modeling of *Agaricus bisporus*, *Cyttaria espinosae* and *Grifola gargal* under hot-air drying and microwave-vacuum drying

**Fig. 1:**
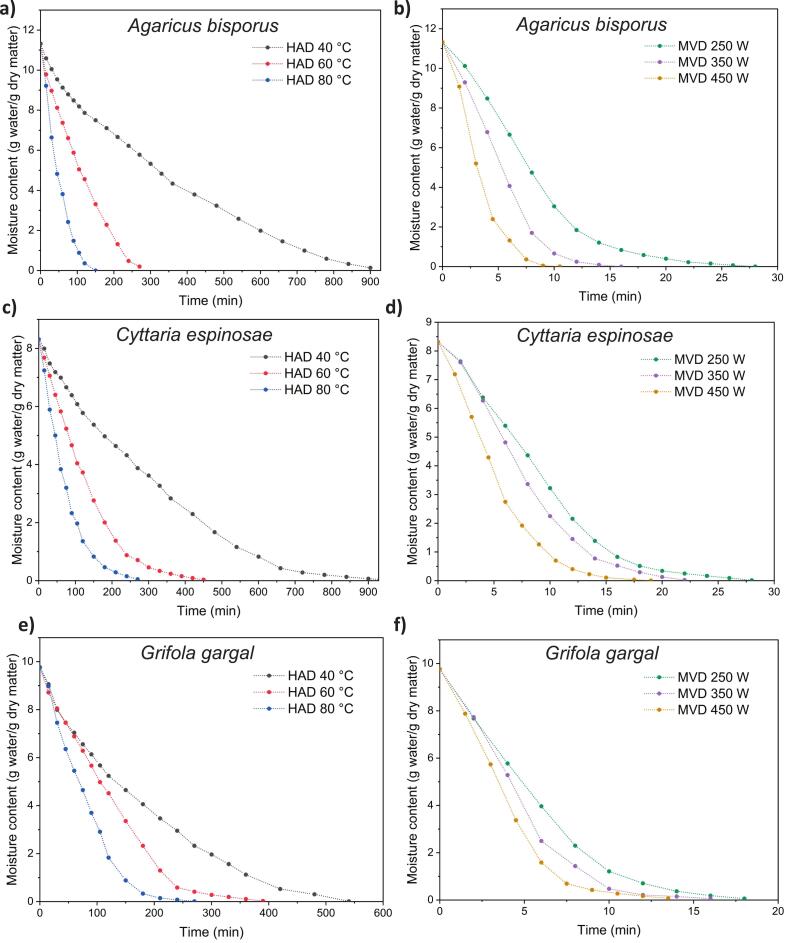
Drying kinetics of (a,b) *Agaricus bisporus*, (c,d) *Cyttaria espinosae* and (e,f) *Grifola gargal* under hot-air drying (HAD) and microwave vacuum drying (MVD).

**Fig. 2:**
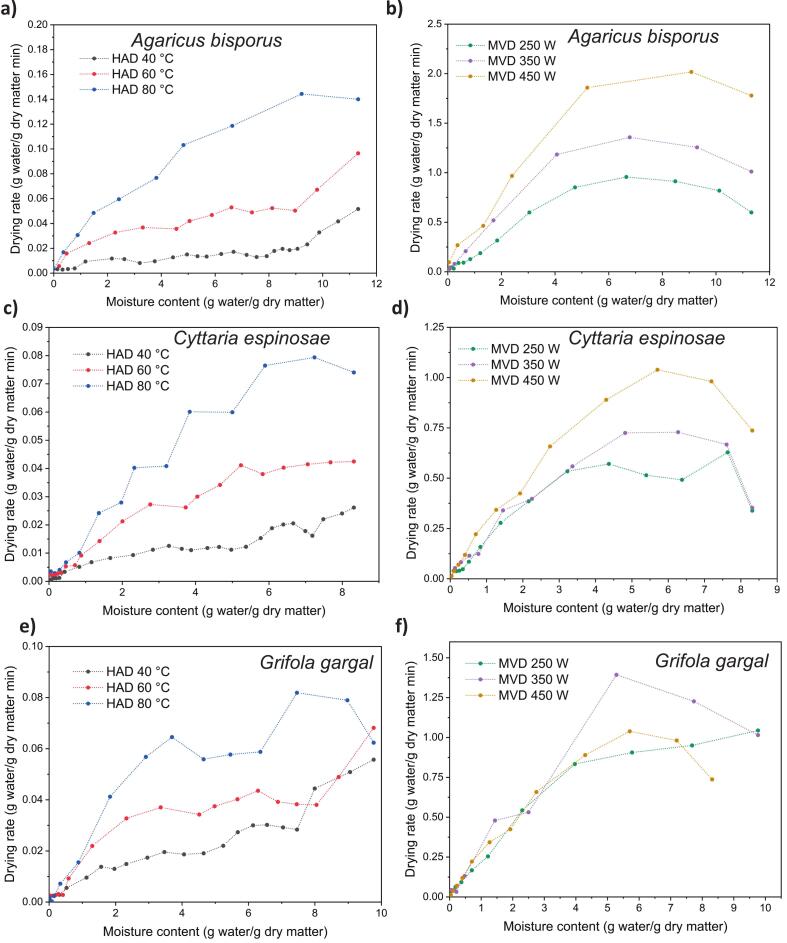
Drying rate curves of (a,b) *Agaricus bisporus*, (c,d) *Cyttaria espinosae* and (e,f) *Grifola gargal* under hot-air drying (HAD) and microwave vacuum drying (MVD).

The moisture-ratio and drying-rate curves obtained for the three mushroom species under hot-air drying (HAD) and microwave–vacuum drying (MVD) (Fig. 1, Fig. 2) showed a short initial stage followed by a predominant falling-rate period, without a prolonged constant-rate phase. This behavior indicates that moisture removal was controlled mainly by internal mass-transfer resistance rather than by surface evaporation alone. At the beginning of drying, surface water and weakly retained water were removed rapidly because of the high moisture and vapor-pressure gradients. As drying progressed, the proportion of more strongly bound water increased and the internal moisture gradient decreased, causing the drying rate to decline. Under HAD, this reduction may also have been intensified by progressive matrix shrinkage and structural collapse, which can reduce pore accessibility, increase transport-path tortuosity, and restrict the diffusion of water from the inner tissue toward the surface. In contrast, the steeper moisture-ratio decline observed under MVD is consistent with volumetric energy deposition, internal vapor generation, and pressure-driven moisture transport under vacuum, which shorten the duration of the diffusion-controlled stage and accelerate water removal (See Table 1.).

**Table 1:** Estimated Page model parameters (k and n) and statistical indicators (R^2^, RMSE and χ^2^) for the drying of three mushroom species under hot-air drying (HAD) and microwave vacuum drying (MVD).

Drying method	k (min^−1^)	n (−)	R^2^	RMSE	χ^2^ (×10^−5^)
*Agaricus bisporus*					
HAD 40 °C	0.00235	1.037	0.9828	0.0211	7.041
HAD 60 °C	0.00274	1.243	0.9879	0.0271	10.308
HAD 80 °C	0.00600	1.314	0.9958	0.022	6.884
MVD 250 W	0.03014	1.634	0.9991	0	0.818
MVD 350 W	0.04766	1.749	0.9982	0.0315	3.266
MVD 450 W	0.12176	1.673	0.9987	0.0376	2.617
*Cyttaria espinosae*					
HAD 40 °C	0.00173	1.114	0.9929	0.0242	4.363
HAD 60 °C	0.00178	1.292	0.9988	0.0137	0.711
HAD 80 °C	0.00497	1.230	0.9992	0.0092	0.668
MVD 250 W	0.02298	1.643	0.9982	0.0133	1.485
MVD 350 W	0.02546	1.731	0.9995	0.0102	0.253
MVD 450 W	0.07277	1.498	0.9993	0.0098	0.499
*Grifola gargal*					
HAD 40 °C	0.00429	1.043	0.9926	0.0176	3.720
HAD 60 °C	0.00132	1.385	0.9923	0.0240	5.845
HAD 80 °C	0.00157	1.448	0.9940	0.0202	4.375
MVD 250 W	0.07543	1.438	0.9980	0.0201	2.586
MVD 350 W	0.07038	1.654	0.9980	0.0090	2.657
MVD 450 W	0.09481	1.640	0.9984	0.0090	2.127

k = drying constant; n = model exponent; R^2^ = coefficient of determination; RMSE = root mean square error; χ^2^ = chi-square.

A physicochemical interpretation of the Page parameters (*k*,n) provides further mechanistic insight. Although the Page model is semi-empirical, k integrates the combined effect of driving forces (thermal and vapor-pressure gradients), heating efficiency, and effective transport properties, acting as a global descriptor of the moisture-removal rate. The increase in k under MVD is consistent with intensified heat–mass coupling due to volumetric heating and internal vapor generation, which reduces diffusion limitations and accelerates moisture migration. The exponent n modulates kinetic curvature and is often associated with deviations from a simple exponential (*n* = 1), reflecting changes in mechanism and/or in effective properties during drying (e.g., MR-dependent effective diffusivity, shrinkage, surface hardening, or pore development) (Bozkır, 2006). This interpretation aligns with critical reviews of thin-layer drying models, in which Page is frequently highlighted for its ability to “absorb” the structural complexity of heterogeneous foods while enabling statistically robust comparisons across process conditions (Erbay & Icier, 2010). In this context, n tended to be higher under MVD than under HAD for *A. bisporus* (1.04–1.31 in HAD vs 1.63–1.75 in MVD), consistent with a more intense onset and a more non-linear kinetic response typically observed in microwave–vacuum drying (M. Zhang et al., 2006).

The species-dependent shapes of the drying curves indicate that the internal resistance to moisture migration differed among the three fungal matrices. Under HAD, *Agaricus bisporus* showed a comparatively clear acceleration as temperature increased, with k rising from 0.00235 to 0.00600 and n from 1.037 to 1.314 between 40 and 80 °C. This response suggests that the stronger thermal and vapor-pressure gradients generated at 80 °C partially overcame the increasing internal resistance associated with moisture depletion and tissue shrinkage. By contrast, *Cyttaria espinosae* exhibited the lowest k values under HAD (0.00173–0.00497), particularly at 40 and 60 °C, indicating a more persistent restriction to internal water migration. This behavior may be associated with species-specific differences in tissue compactness, cell-wall organization, and the relative distribution of free and bound water.

The kinetic response of *Grifola gargal* was more complex because k did not increase monotonically with temperature, whereas n increased from 1.043 to 1.448. Therefore, the two Page parameters should be interpreted jointly rather than considering k as an isolated drying-rate coefficient. The higher n values at 60 and 80 °C indicate a stronger curvature of the drying process and suggest that temperature modified the relative contribution of internal diffusion, structural rearrangement, and shrinkage during moisture removal. Collectively, these differences show that the drying behavior was governed not only by the imposed temperature but also by the distinct architecture and water-binding characteristics of each mushroom matrix.

Under MVD, the marked increase in k across all species reflected a substantial reduction in the apparent internal resistance to moisture transport, although the magnitude of this response remained dependent on the fungal matrix. *Grifola gargal* exhibited comparatively high k values even at 250 and 350 W, suggesting that its tissue structure provided relatively accessible pathways for the evacuation of internally generated vapor. In contrast, *Cyttaria espinosae* retained the lowest k values among the three species at each microwave power, indicating that its intrinsic resistance to moisture migration was only partially overcome by volumetric heating. *Agaricus bisporus* showed the strongest response to increasing microwave power, with k rising from 0.03014 at 250 W to 0.12176 at 450 W. Unlike HAD, in which moisture transport during the falling-rate period is governed predominantly by internal diffusion toward a progressively drier surface, MVD combines moisture diffusion with pressure-driven vapor flow generated within the sample. The rapid formation and evacuation of internal vapor may preserve transport pathways and partially counteract pore closure caused by matrix shrinkage, thereby accelerating moisture removal (Giri & Prasad, 2007; González-Cavieres et al., 2021).

Because dimensional changes and internal moisture profiles were not measured directly, the contribution of matrix shrinkage and effective water diffusion could not be quantified. These mechanisms are therefore proposed as physicochemically plausible interpretations of the species-dependent drying curves and should be confirmed through future diffusivity and shrinkage measurements.

### 3.2 Effect of drying method on color parameters and free sugar composition

The drying strategy induced species-dependent shifts in instrumental color (L*, a*, b*, ΔE, and browning index, BI) (Table 2), consistent with the coupled contributions of enzymatic browning (PPO-catalyzed oxidation of phenolics during the early stages when oxygen is still available), non-enzymatic browning (Maillard-type pathways driven by reducing sugars reacting with amino compounds), and microstructural reconfiguration (shrinkage/collapse versus porosity development) that alters light scattering and thus the apparent chromatic response. These concurrent mechanisms are widely documented for mushroom tissues and for microwave-based dehydration, where dielectric heterogeneity and rapid internal heating can amplify local thermal gradients even under reduced pressure(Dehghannya & Habibi‐Ghods, 2025; Duan et al., 2016).

**Table 2:** Color parameters of fresh and dried mushrooms processed by hot-air drying (HAD) and microwave vacuum drying (MVD).

Species / Drying method	L*	a*	b*	C*	h°	∆*E*	BI value
	*Agaricus bisporus*						
Fresh	72.95 ± 0.06 ᵃ	2.65 ± 0.02 ᵉ	12.19 ± 0.04 ᵍ	12.47 ± 0.04 ᵍ	77.73 ± 0.05 ᶜ	–	–
HAD 40 °C	65.01 ± 0.07 ᶠ	3.42 ± 0.05 ᵇᶜ	13.93 ± 0.01 ᶠ	14.34 ± 0.01 ᶠ	76.19 ± 0.20 ᵈ	8.16 ± 0.08 ᶜ	27.53 ± 0.08 ᵃ
HAD 60 °C	63.17 ± 0.05 ᵍ	3.77 ± 0.12 ᵃᵇ	17.96 ± 0.06 ᶜ	18.35 ± 0.04 ᶜ	78.13 ± 0.41 ᶜ	11.41 ± 0.09 ᵉ	37.22 ± 0.07 ᶜ
HAD 80 °C	69.58 ± 0.08 ᵇ	2.98 ± 0.03 ᵈᵉ	14.89 ± 0.05 ᵉ	15.19 ± 0.05 ᵉ	78.68 ± 0.11 ᵇᶜ	4.33 ± 0.06 ᵃ	26.76 ± 0.13 ᵃ
MVD 250 W	68.25 ± 0.08 ᶜ	3.50 ± 0.05 ᵇᶜ	16.56 ± 0.06 ᵈ	16.93 ± 0.05 ᵈ	78.08 ± 0.18 ᶜ	6.47 ± 0.10 ᵇ	31.04 ± 0.12 ᵇ
MVD 350 W	66.29 ± 0.12 ᵈ	3.31 ± 0.20 ᶜᵈ	19.94 ± 0.13 ᵇ	20.21 ± 0.10 ᵇ	80.56 ± 0.62 ᵃ	10.24 ± 0.06 ᵈ	38.74 ± 0.03 ᵈ
MVD 450 W	65.87 ± 0.08 ᵉ	4.03 ± 0.17 ᵃ	21.52 ± 0.05 ᵃ	21.89 ± 0.02 ᵃ	79.38 ± 0.45 ᵃᵇ	11.79 ± 0.09 ᶠ	43.26 ± 0.16 ᵉ
	*Cyttaria espinosae*						
Fresh	66.12 ± 0.12 ᵃ	21.52 ± 0.01 ᵃ	43.87 ± 0.01 ᵃ	48.86 ± 0.00 ᵃ	63.87 ± 0.02 ᶠ	–	–
HAD 40 °C	65.21 ± 0.01 ᵇ	11.44 ± 0.06 ᵍ	35.48 ± 0.06 ᵉ	37.28 ± 0.04 ᵈ	72.13 ± 0.12 ᵃ	5.77 ± 0.06 ᵃ	88.49 ± 0.13 ᵃ
HAD 60 °C	65.19 ± 0.01 ᵇ	13.59 ± 0.05 ᵈ	38.65 ± 0.08 ᶜ	40.97 ± 0.06 ᶜ	70.63 ± 0.09 ᵇ	7.96 ± 0.06 ᵇ	100.87 ± 0.18 ᶜ
HAD 80 °C	62.69 ± 0.07 ᶜ	13.99 ± 0.06 ᶜ	38.40 ± 0.04 ᵈ	40.87 ± 0.02 ᶜ	69.98 ± 0.09 ᶜ	18.52 ± 0.07 ᶠ	106.14 ± 0.09 ᵈ
MVD 250 W	59.11 ± 0.05 ᵈ	15.52 ± 0.08 ᵇ	39.65 ± 0.08 ᵇ	42.58 ± 0.05 ᵇ	68.63 ± 0.14 ᵉ	11.34 ± 0.78 ᶜ	122.20 ± 0.20 ᵉ
MVD 350 W	59.26 ± 0.08 ᵈ	13.12 ± 0.06 ᶠ	34.42 ± 0.02 ᶠ	36.84 ± 0.01 ᵉ	69.13 ± 0.09 ᵈ	14.08 ± 0.11 ᵈ	99.34 ± 0.19 ᵇ
MVD 450 W	58.67 ± 0.06 ᵉ	13.37 ± 0.05 ᵉ	33.70 ± 0.08 ᵍ	36.25 ± 0.06 ᶠ	68.35 ± 0.10 ᵉ	15.62 ± 0.17 ᵉ	98.48 ± 0.21 ᵇ
	*Grifola gargal*						
Fresh	68.46 ± 0.17 ᵃ	1.94 ± 0.06 ᵍ	22.63 ± 0.02 ᶠ	22.72 ± 0.03 ᵈ	85.09 ± 0.14 ᵃ	–	–
HAD 40 °C	65.82 ± 0.08 ᵇ	4.54 ± 0.06 ᶠ	27.06 ± 0.01 ᵉ	27.44 ± 0.00 ᶜ	80.47 ± 0.12 ᵇ	13.14 ± 0.02 ᶜ	56.71 ± 0.06 ᵃ
HAD 60 °C	63.88 ± 0.08 ᶜ	6.01 ± 0.06 ᵉ	27.71 ± 0.07 ᵈ	28.35 ± 0.06 ᶜ	77.76 ± 0.15 ᶜ	9.53 ± 0.02 ᵃ	62.37 ± 0.16 ᵇ
HAD 80 °C	55.49 ± 0.13 ᵉ	11.39 ± 0.08 ᵃ	31.88 ± 0.07 ᵃ	33.85 ± 0.04 ᵃ	70.33 ± 0.17 ᵍ	9.91 ± 0.05 ᵇ	96.82 ± 0.16 ᵉ
MVD 250 W	61.29 ± 1.08 ᵈ	7.84 ± 0.06 ᶜ	29.05 ± 0.03 ᶜ	30.09 ± 0.04 ᵇ	74.89 ± 0.09 ᵉ	10.15 ± 0.10 ᵇ	71.95 ± 1.54 ᶜ
MVD 350 W	59.99 ± 0.02 ᵈ	9.40 ± 0.04 ᵇ	31.05 ± 0.04 ᵇ	32.44 ± 0.03 ᵃ	73.16 ± 0.09 ᶠ	14.38 ± 0.05 ᵈ	81.99 ± 0.10 ᵈ
MVD 450 W	54.99 ± 0.12 ᵉ	6.74 ± 0.22 ᵈ	28.01 ± 0.15 ᶜ	29.68 ± 0.10 ᵇ	76.87 ± 0.48 ᵈ	15.01 ± 0.05 ᵉ	80.91 ± 0.12 ᵈ

For *Agaricus bisporus*, HAD at higher temperature preserved lightness more effectively (HAD 80 °C: L* = 69.58; ΔE = 4.33; BI = 26.76), whereas increasing MVD power progressively intensified darkening/browning (MVD 450 W: L* = 65.87; ΔE = 11.79; BI = 43.26) together with higher b* and chroma (b* = 21.52; C* = 21.89), indicating stronger yellow–brown development at the highest microwave load. From a mechanistic standpoint, vacuum operation can lower the boiling point and limit oxidative routes; however, high microwave power may promote localized overheating (“hot spots”) due to spatially variable dielectric properties and water distribution, accelerating Maillard chemistry and pigment transformations despite shorter residence times (Dumpler & Moraru, 2023; Hemmler et al., 2018).

In *Cyttaria espinosae*, a naturally highly chromatic matrix (fresh a* = 21.52; b* = 43.87), both HAD and MVD markedly modified redness/yellowness and increased BI, albeit along distinct trajectories: HAD primarily reduced a* while preserving relatively high L*, whereas MVD led to lower L* and, at 250 W, the highest BI (122.20) despite elevated b*. This behavior is characteristic of pigmented tissues in which microwave–vacuum conditions promote a shift from simple chromophore loss toward net darkening through concurrent pigment transformation and early-stage non-enzymatic browning, particularly under spatially heterogeneous thermal histories (Dehghannya & Habibi‐Ghods, 2025; Duan et al., 2016). Similarly, *Grifola gargal* exhibited pronounced browning under both drying routes, with HAD 80 °C yielding the highest BI (96.82) and MVD sustaining elevated BI at higher powers (81–82), together with reduced L* and increased a*–b* coordinates. In protein-rich fungal matrices, such responses are consistent with thermally driven melanoidin formation once water activity enters the kinetic window favoring Maillard pathways, governed by the coupled effects of temperature, exposure time, and moisture state (El Hosry et al., 2025; Hemmler et al., 2018).

Free sugar profiles (Table 3) provide a chemically coherent basis to interpret the color outcomes while demonstrating that “sugar concentration” under drying is not purely a mass-balance artifact. Drying can increase extractable sugars by disrupting cell walls and improving solvent accessibility yet decrease them through consumption in browning reactions and thermal degradation. This duality is particularly relevant in microwave-based processes, where intensified internal heating can enhance both liberation and reaction of reducing sugars (Dehghannya & Habibi‐Ghods, 2025; Hemmler et al., 2018).

**Table 3:** Sugar content (g/100 g d.b.) in fresh and dried mushrooms processed by hot-air drying (HAD) and microwave vacuum drying (MVD).

Species / Sugar	Fresh	HAD 40 °C	HAD 60 °C	HAD 80 °C	MVD 250 W	MVD 350 W	MVD 450 W
*Agaricus bisporus*							
Arabinose	nd	nd	nd	nd	nd	nd	nd
Fructose	nd	nd	nd	nd	nd	nd	nd
Glucose	12.28 ± 0.42 ᵉ	14.98 ± 0.17 ᶜ	12.96 ± 0.17 ᵈᵉ	13.97 ± 0.32 ᶜᵈ	22.59 ± 0.67 ᵃ	16.96 ± 0.33 ᵇ	16.65 ± 0.48 ᵇ

*Cyttaria espinosae*							
Arabinose	4.79 ± 0.18 ᶜ	6.75 ± 0.29 ᵇ	7.13 ± 0.30 ᵇ	4.97 ± 0.19 ᶜ	10.79 ± 0.51 ᵃ	7.23 ± 0.03 ᵇ	6.55 ± 0.33 ᵇ
Fructose	3.61 ± 0.03 ᵃ	2.34 ± 0.08 ᶜ	2.26 ± 0.09 ᶜᵈ	2.03 ± 0.02 ᵈ	3.00 ± 0.07 ᵇ	2.07 ± 0.07 ᵈ	2.05 ± 0.07 ᵈ
Glucose	10.06 ± 0.45 ᵇ	11.77 ± 0.16 ᵃ	8.68 ± 0.42 ᶜᵈ	7.72 ± 0.28 ᵈᵉ	9.49 ± 0.46 ᵇᶜ	7.47 ± 0.15 ᵉ	7.12 ± 0.26 ᵉ

*Grifola gargal*							
Arabinose	nd	nd	nd	nd	nd	nd	nd
Fructose	nd	nd	nd	nd	nd	nd	nd
Glucose	2.93 ± 0.04 ᵃ	1.55 ± 0.04 ᵈ	1.35 ± 0.05 ᵈ	2.00 ± 0.08 ᵇ	1.79 ± 0.08 ᶜ	1.54 ± 0.04 ᵈ	1.51 ± 0.05 ᵈ

Across species, drying modulated free sugar profiles in a manner consistent with the competing effects of matrix disruption and Maillard consumption. In *A. bisporus*, only glucose was detected, with MVD at 250 W producing a pronounced increase (22.59 g/100 g d.b.) relative to fresh material (12.28 g/100 g d.b.), followed by lower levels at 350–450 W (16.7–17.0 g/100 g d.b.), indicative of maximal structural solubilization at moderate power and partial glucose consumption at higher microwave loads, in agreement with the concurrent rise in BI and ΔE. In *C. espinosae*, arabinose, fructose, and glucose were quantified, with arabinose peaking under MVD 250 W (10.79 g/100 g d.b. vs 4.79 fresh) while fructose and glucose declined with increasing process intensity, consistent with enhanced liberation of highly Maillard-reactive pentoses under moderate MVD and progressive sugar consumption at higher severity. In *G. gargal*, only glucose was detected and decreased after drying (2.93 → 1.35–2.00 g/100 g d.b.), supporting net sugar transformation during browning despite modest initial availability. Collectively, these trends reflect a balance between microwave-induced matrix disruption and thermally driven Maillard pathways, strongly governed by thermal history and reactant availability, with pentose release under moderate MVD further amplifying browning propensity in amino-rich fungal matrices (Bork et al., 2024; El Hosry et al., 2025; Hemmler et al., 2018; Lund & Ray, 2017).

Values are expressed as mean ± standard deviation of three determinations. Different superscript letters within the same species and column indicate significant differences among drying treatments according to one-way ANOVA followed by Tukey's test (*p* < 0.05). Fresh samples were used as the reference for ΔEcalculations and were therefore not included in the statistical comparison for this parameter.

Overall, the joint evolution of color parameters and free sugars indicates that drying responses are governed by the balance between matrix disruption and Maillard-driven consumption, modulated by process intensity and species composition. Although MVD may limit oxidative discoloration, it can simultaneously enhance the release of reactive sugars and generate localized thermal gradients, increasing susceptibility to non-enzymatic browning at higher powers. These results emphasize that visual quality reflects underlying structural and chemical changes in the matrix, providing the basis for analyzing drying-induced modifications in β-glucan organization and composition.

### 3.3 Interspecies differences in native glucan content

The enzymatic quantification of glucan fractions in fresh fruiting bodies (Table 4) reveals marked interspecies differences that establish a compositional baseline for interpreting subsequent drying-induced structural and biological effects. Fungal cell walls are principally composed of polysaccharides such as α- and β-glucans, together with chitin and associated glycoproteins, which vary in relative abundance across taxa and contribute to mechanical strength and physiological function (Ruiz-Herrera & Ortiz-Castellanos, 2019). In this context, *Grifola gargal* exhibits the highest β-glucan content (55.02 ± 3.61 g/100 g d.b.), consistent with polypore-type fungi being rich in structural β-glucans that form microfibrillar scaffolds within the cell wall. *Cyttaria espinosae* shows an intermediate but substantial β-glucan level (31.00 ± 1.13 g/100 g d.b.) together with a comparatively elevated α-glucan fraction (7.22 ± 0.31 g/100 g d.b.), suggesting a more heterogeneous polysaccharide matrix where α-linked glucans contribute non-structural or matrix roles (Flores et al., 2024). In contrast, *Agaricus bisporus* presents a much lower β-glucan content (9.42 ± 1.40 g/100 g d.b.), in agreement with reported ranges for edible mushrooms, where button mushrooms typically show relatively modest β-glucan proportions compared to other taxa (Belobrajdic et al., 2024). These intrinsic compositional differences are relevant because β-glucan abundance alone does not dictate functionality; rather, the relative balance between α- and β-linked glucans and their embedding within the native cell-wall network influence extractability, supramolecular rearrangement upon drying, and ultimately biological presentation. In this framework, the high β-glucan density of *G. gargal* provides a favorable starting point for structural organization, whereas the mixed α/β composition of *C. espinosae* likely contributes to the broader distribution of supramolecular states observed after processing. Importantly, establishing these fresh-matrix baselines allows the effects of HAD and MVD discussed in 3.3, 3.4, 3.5 to be interpreted primarily as consequences of drying-driven reorganization rather than simple differences in glucan availability, reinforcing the central premise that process-induced structuring—rather than content alone—governs the functional performance of mushroom-derived β-glucans.

**Table 4:** α-, β-, and total glucan contents (g/100 g dry basis) determined enzymatically in fresh fruiting bodies of *Agaricus bisporus*, *Cyttaria espinosae*, and *Grifola gargal*.

Species / Glucans	α-glucans	β-glucans	Total glucans
*Agaricus bisporus*	0.83 ± 0.25 ^b^	9.42 ± 1.40 ^c^	10.25 ± 1.15 ^c^
*Cyttaria espinosae*	7.22 ± 0.31 ^a^	31.00 ± 1.13 ^b^	38.22 ± 1.14 ^b^
*Grifola gargal*	0.48 ± 0.21 ^b^	55.02 ± 3.61 ^a^	55.50 ± 3.59 ^a^

Values are expressed as mean ± standard deviation of three determinations. Different superscript letters within the same column indicate significant differences among mushroom species according to one-way ANOVA followed by Tukey's multiple-comparison test (*p* < 0.05).

### 3.4 Thermal transitions and supramolecular organization of mushroom β-glucans

**Fig. 3:**
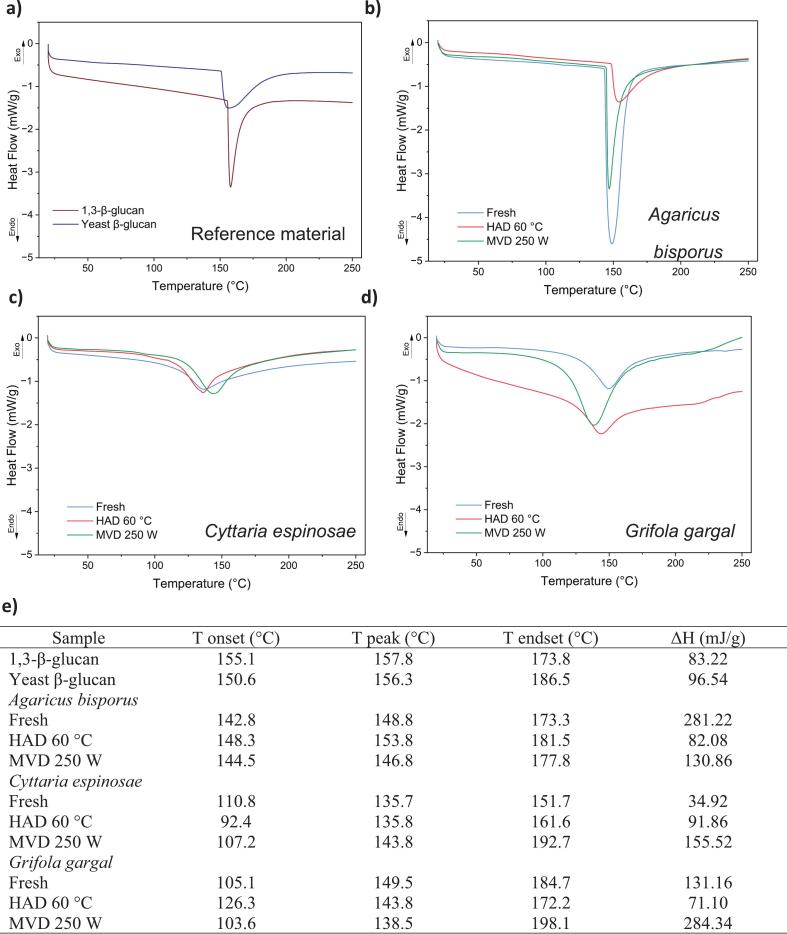
Differential scanning calorimetry (DSC) thermograms of β-glucans extracted from edible mushrooms processed under different drying methods. (a) Reference β-glucans; (b) *Agaricus bisporus* β-glucans; (c) *Cyttaria espinosae* β-glucans; and (d) *Grifola gargal* β-glucans. (e) Summary panel showing onset, peak, and endset temperatures, as well as ΔH values for all extracted β-glucan samples.

The DSC thermograms (Fig. 3) exhibit a dominant endothermic event spanning approximately 110–200 °C, consistent with a cooperative transition governed by dehydration and disruption of hydrogen-bond networks and supramolecular order, rather than by changes in the fundamental chemical identity of the polymer (Mlčoch & Kučerík, 2013). For β-(1 → 3)-glucans such as curdlan, endotherms in this temperature domain have been associated with order–disorder processes coupled to bound-water release and weakening of intra−/interchain hydrogen bonding, with strong sensitivity to hydration history and helix packing (Puertas et al., 2014). Accordingly, the reference materials (1,3-β-glucan and yeast β-glucan, Fig. 3a) concentrate their transitions near 156–158 °C with moderate ΔH, providing a benchmark indicating that shifts in Tonset/Tpeak/Tendset—and particularly changes in ΔH—primarily report the extent and stability of supramolecular organization (and strongly associated water) participating in the transition, rather than the emergence of new chemical functionalities (Xu et al., 2013).

In *Agaricus bisporus* (Fig. 3b), the fresh extract displays the highest ΔH across the dataset, indicative of a highly cooperative transition involving a substantial fraction of ordered domains and/or a large contribution from tightly bound water requiring elevated energy input for release and structural relaxation (Gun'ko et al., 2017). After drying, the thermal signature converges toward less energetic transitions: HAD at 60 °C increases Tonset/Tpeak but markedly decreases ΔH, suggesting a shift toward thermally more stable yet less extensive cooperative domains, as expected for more dehydrated, compact networks in which a smaller fraction of polymer–water assemblies participates in the endotherm (Mlčoch & Kučerík, 2013). MVD at 250 W yields an intermediate response (ΔH higher than HAD with comparable characteristic temperatures), consistent with volumetric heating preserving part of the supramolecular organization without reaching the same degree of prolonged dehydration/compaction associated with convective drying (Tegopoulos et al., 2024). This pattern aligns with literature emphasizing that hydration state and helical packing control both the apparent transition temperature and enthalpy in polysaccharide systems by modulating hydrogen-bond cooperativity and the mass fraction of “ordered” material undergoing the transition (Hatakeyama et al., 2016).

For *Cyttaria espinosae* and *Grifola gargal*, the drying route more clearly modulates thermal stability (Tendset) and cooperative energy (ΔH). In *Cyttaria*, the fresh material exhibits a comparatively weak event (low ΔH) terminating at lower temperature, whereas drying—especially MVD at 250 W—broadens the transition toward higher temperatures (substantially elevated Tendset) and increases ΔH, consistent with the formation and/or preservation of more thermally stable and heterogeneous supramolecular assemblies (i.e., a wider distribution of ordered populations and bound-water environments) (Marelli et al., 2025). In *Grifola*, HAD at 60 °C decreases ΔH, indicating reduced cooperativity and/or a diminished proportion of ordered domains participating in the transition, while MVD at 250 W maximizes ΔH and shifts the end of the event to higher temperature, supporting a scenario in which microwave-vacuum processing favors more cooperative supramolecular reorganization (e.g., enhanced helical aggregation and order–disorder contrast) without the same structural penalties associated with extended thermal exposure (Q. Zhang et al., 2024). These behaviors are consistent with recent reports highlighting that β-glucans of similar overall chemistry can exhibit distinct thermal fingerprints driven by differences in conformation, packing, and polymer–water coupling (Mlčoch & Kučerík, 2013). These thermal differences were subsequently interpreted together with the morphological and spectroscopic results to distinguish drying-induced supramolecular reorganization from changes in the chemical identity of the β-glucans.

### 3.5 Microstructural, spectroscopic, and integrated structural analysis of β-glucans

**Fig. 4:**
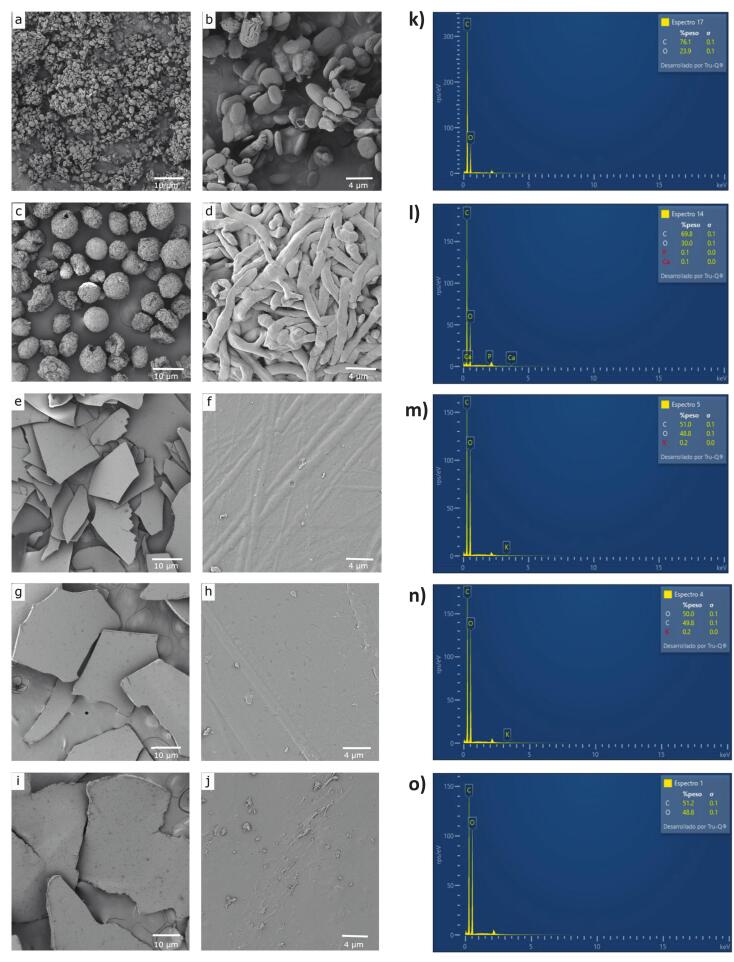
SEM micrographs and corresponding EDS spectra of β-glucans extracted from the studied mushroom samples. SEM images show: (a–b) 1,3-β-glucan reference; (c–d) yeast β-glucan; (e–f) β-glucans extracted from fresh *Cyttaria espinosae*; (g–h) β-glucans extracted from *C. espinosae* dried by convective drying at 60 °C; and (i–j) β-glucans extracted from *C. espinosae* dried by vacuum drying at 250 W. The EDS spectra (right panels) correspond to each SEM image and confirm the characteristic carbon–oxygen-dominant composition of the extracted β-glucan materials.

The SEM micrographs (Fig. 4) indicate that extraction yields materials whose morphologies are strongly dependent on biological origin and, in the case of *Cyttaria espinosae*, are also sensitive to the drying route. The reference materials exhibit typical architectures: 1,3-β-glucan shows irregular/particulate aggregates, whereas yeast β-glucan displays particulate domains and more “cell-like” structures associated with cell-wall β-glucans, often described as porous/particulate microstructures derived from the glucan scaffold (Guo et al., 2019; Hatakeyama et al., 2016). In *C. espinosae*, β-glucan extracted from fresh material tends to organize into sheet−/plate-like structures with relatively continuous surfaces, consistent with post-extraction drying collapse and re-aggregation via hydrogen bonding during water removal (Nsor-Atindana et al., 2017). When routes are compared, convective drying (HAD 60 °C) is associated with more compact plates and flatter surfaces, compatible with shrinkage and densification driven by slow, prolonged dehydration; by contrast, microwave–vacuum drying (MVD 250 W) retains greater surface heterogeneity (fractures/micro-irregularities), consistent with volumetric heating and the formation of internal vapor gradients that promote micro-fracturing and/or fine porosity—an effect widely reported for food matrices processed by MVD relative to HAD (Mlčoch & Kučerík, 2013; Q. Zhang et al., 2025). These morphological differences were consistent with the DSC responses observed for the corresponding *C. espinosae* samples. The compact and flattened morphology produced by HAD was associated with matrix densification and closer polymer association during prolonged dehydration. In contrast, the heterogeneous and fractured morphology observed after MVD coincided with a broader thermal transition, a higher T_endset_, and an increased (ΔH). The combined evidence suggests that MVD produced more heterogeneous but thermally cooperative β-glucan assemblies, whereas HAD favored a more compact organization resulting from slower dehydration, contraction, and polymer re-aggregation.

In parallel, the EDS spectra (right panels, Fig. 4) confirm a carbon- and oxygen-dominant composition in all cases, with minor inorganic traces (e.g., K/Ca/P) attributable to mineral residues from the matrix or remnant salts, supporting the conclusion that the observed differences are primarily microstructural rather than reflecting differential “mineralization” of the extracted fraction (Yesiltas et al., 2018).

**Fig. 5:**
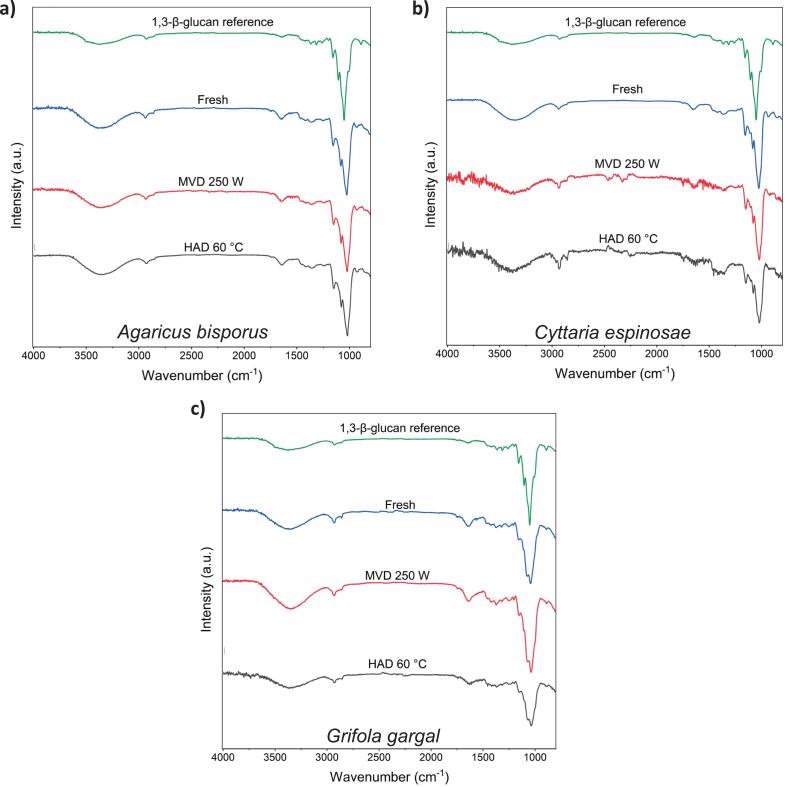
FTIR spectra of mushroom extracts from *Agaricus bisporus*, *Cyttaria espinosae*, and *Grifola gargal* under different drying conditions (fresh, HAD 60 °C, and MVD 250 W).

The FTIR spectra (Fig. 5) are collectively consistent with preservation of the polymer's chemical identity across drying routes, with changes more plausibly interpreted as conformational and hydration rearrangements (Wu et al., 2016). Bands dominated by O—H vibrations (broad region 3600–3200 cm^−1^), C—H (2920 cm^−1^), contributions from water/proteins around 1650–1540 cm^−1^ when present, and the “carbohydrate region” (1200–950 cm^−1^) associated with C–O/C–O–C stretching of the polysaccharide backbone are observed (Baeva et al., 2019; L. Chen et al., 2024). Particularly informative was the persistence of the band near 890–894 cm^−1^, assigned to the anomeric deformation characteristic of β-linked glucans, which supports retention of the β-glucan motif across drying conditions. Meanwhile, subtle variations in the intensity and width of the O—H region and the carbohydrate region at 1200–950 cm^−1^ were consistent with changes in polymer–water interactions, hydrogen-bond organization, and supramolecular packing rather than with the formation of new chemical functionalities (Xu et al., 2013).

The combined DSC, SEM, and FTIR results indicate that the drying treatments primarily modified the supramolecular organization of the extracted β-glucans rather than their fundamental chemical identity. Across species and treatments, the persistence of the characteristic β-glucan band near 890–894 cm^−1^, together with the absence of new diagnostic absorption bands, confirmed preservation of the β-linked polysaccharide backbone. Therefore, the treatment-dependent changes in transition temperatures and ΔH are more consistently attributed to differences in hydrogen-bond cooperativity, bound-water environments, chain association, and molecular packing than to chemical degradation or the formation of new functional groups.

This relationship was particularly evident for *C. espinosae*, for which comparable DSC, SEM, and FTIR results were available. The compact plate-like morphology observed after HAD was consistent with drying-induced densification and redistribution of hydrogen-bonded domains, whereas the fractured and heterogeneous morphology produced by MVD coincided with a broader and more energetic thermal transition. At the same time, the preservation of the characteristic FTIR fingerprint, together with subtle variations in the O—H and carbohydrate regions, supported changes in polymer–water interactions and supramolecular packing. Thus, the three techniques provide complementary evidence that HAD favored more compact and collapsed assemblies, whereas MVD generated more heterogeneous and thermally cooperative structures without altering the underlying β-glucan chemistry.

### 3.6 Cytocompatibility of β-glucans obtained under different drying conditions

**Fig. 6:**
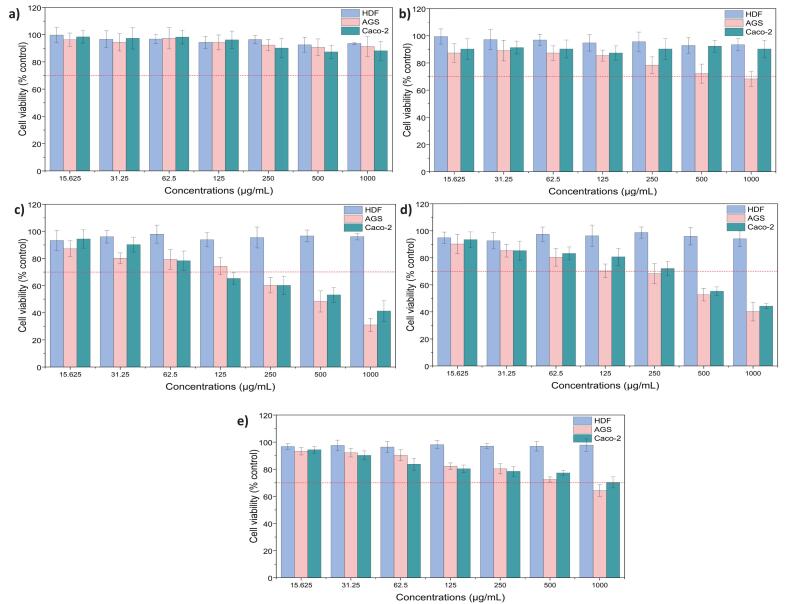
Cell viability of HDF, AGS, and Caco-2 cells exposed to β-glucan extracts. (a) 1,3-β-glucans; (b) yeast β-glucan; (c) *C. espinosae* β-glucans obtained after microwave-vacuum drying at 250 W; (d) *C. espinosae* β-glucans obtained after convective drying at 60 °C; (e) fresh *Cyttaria espinosae* β-glucans. The red dashed line indicates the 70% threshold for cytotoxicity according to ISO 10993-5. (For interpretation of the references to color in this figure legend, the reader is referred to the web version of this article.)

Cell viability of human dermal fibroblasts (HDF) and gastrointestinal cancer-derived cell lines (AGS and Caco-2) was evaluated after exposure to β-glucan references and *Cyttaria espinosae* β-glucan extracts (15.625–1000 μg/mL) (Fig. 6). The ISO 10993-5 cytotoxicity threshold (70% viability) is indicated by the red dashed line (International Organization for Standardization, 2017). Across all treatments, HDF viability remained consistently high (93–99%), supporting overall cytocompatibility of the extracts and indicating that neither drying route introduced overt toxicity toward normal cells within the tested range (Muthuramalingam et al., 2019).

Reference β-glucans produced limited biological impact in cancer cells. 1,3-β-glucan maintained viability above the ISO threshold in all cell types across all concentrations, showing only minor decreases at high doses. Yeast β-glucan displayed a clearer dose dependence in AGS, crossing the 70% threshold only at the highest concentration (68.25 ± 5.40% at 1000 μg/mL), while Caco-2 remained largely unaffected (87–92%). These controls set a baseline consistent with β-glucans being broadly biocompatible materials whose antiproliferative effects are modest unless specific structural features enhance cell interactions (Noorbakhsh Varnosfaderani et al., 2024).

In contrast, *C. espinosae* β-glucans exhibited marked, treatment-dependent reductions in AGS and Caco-2 viability, suggesting that processing modulates bioactivity through changes in supramolecular organization and microstructure. The microwave–vacuum dried extract (MVD, 250 W) showed the strongest effect: AGS viability dropped below 70% from 250 μg/mL (60.21 ± 5.60%) and decreased further at higher doses (31.03 ± 4.90% at 1000 μg/mL). Caco-2 was even more sensitive, crossing the ISO threshold already at 125 μg/mL (65.32 ± 4.30%) and reaching 41.27 ± 7.60% at 1000 μg/mL. The convectively dried extract (HAD, 60 °C) also reduced cancer-cell viability but with a slightly shifted onset: AGS crossed below 70% at 250 μg/mL (68.34 ± 7.40%), while Caco-2 remained near/above the threshold up to 250 μg/mL (72.10 ± 5.20%) and fell below it from 500 μg/mL (55.30 ± 3.20%). Fresh β-glucans showed the mildest response, with AGS dropping below 70% only at 1000 μg/mL (64.27 ± 4.31%) and Caco-2 remaining at the threshold even at the highest dose (70.32 ± 4.12%) (Noorbakhsh Varnosfaderani et al., 2024).

When interpreted alongside the structural dataset (3.3, 3.4), the viability trends are best explained by a drying-controlled reorganization of β-glucan assemblies that modulates how the material is “presented” to cancer cells, rather than by chemical modification of the backbone. FTIR indicates preservation of the β-glucan fingerprint, whereas DSC and SEM show that MVD and HAD generate distinct supramolecular states and morphologies. In β-glucans, this level of organization is often decisive for bioactivity because interaction with cells depends on hydration/packing, aggregation and accessible β-(1 → 3) motifs at the biointerface. Under this view, the earlier and stronger reductions in AGS and Caco-2 viability observed for MVD-derived C. espinosae β-glucans are consistent with assemblies that are more interaction-competent (higher effective surface, more defects/heterogeneity, and a broader distribution of ordered populations), while convective drying likely promotes densification and re-aggregation that delays activity until higher concentrations(Meng et al., 2020).

From an anticancer formulation perspective, these results are relevant because they indicate that processing can be used to tune antiproliferative potency without inducing overt toxicity in normal fibroblasts. In practical terms, MVD emerges as a scalable “structuring step” to obtain Cyttaria β-glucans with lower apparent activity thresholds in gastrointestinal cancer-derived models compared with fresh or convectively dried extracts, suggesting added value for development of β-glucan-based bioactive ingredients or adjuvant-type formulations where supramolecular accessibility is critical (Wang et al., 2024). The cell-line dependence (AGS vs Caco-2) further supports that the response is governed by interaction kinetics and cellular phenotype rather than nonspecific toxicity, reinforcing the need to treat β-glucans as organization-dependent anticancer biomacromolecules (Noorbakhsh Varnosfaderani et al., 2024).

## 4 Conclusions

The drying route acted not only as a preservation step but also as a processing variable capable of modifying the organization and biological performance of mushroom β-glucans. The Page model adequately described the predominantly falling-rate drying behavior of the three species (R^2^ = 0.9828–0.9995), while MVD produced substantially higher (k) values (0.02298–0.12176) than HAD (0.00132–0.00600), confirming its greater dehydration efficiency. Native β-glucan contents differed significantly among species, with *Grifola gargal* showing the highest value (55.02 ± 3.61 g/100 g d.b.), followed by *Cyttaria espinosae* (31.00 ± 1.13 g/100 g d.b.) and *Agaricus bisporus* (9.42 ± 1.40 g/100 g d.b.). Although color and free-sugar responses depended on species and process intensity, the integrated DSC, SEM/EDS, and FTIR analyses showed that drying primarily modified thermal cooperativity, polymer–water interactions, and microstructural organization while preserving the characteristic β-glucan spectroscopic fingerprint. This relationship was particularly evident in *C. espinosae*, for which HAD promoted more compact assemblies, whereas MVD generated more heterogeneous and thermally cooperative structures. All *C. espinosae* extracts remained cytocompatible toward HDF cells, with viability between 93 and 99%. However, the MVD-derived extract produced the earliest reduction below 70% viability in Caco-2 cells at 125 μg/mL and in AGS cells at 250 μg/mL, reaching 41.27 ± 7.60% and 31.03 ± 4.90%, respectively, at 1000 μg/mL. Collectively, these results support MVD as an effective strategy for accelerating dehydration and tailoring β-glucan supramolecular organization without evident alteration of its chemical backbone. Further studies should determine molecular-weight distribution and assembly state and confirm the observed biological responses using mechanistic and physiologically representative models.

## CRediT authorship contribution statement

**Ociel Muñoz-Fariña:** Writing – review & editing, Supervision, Resources, Methodology, Investigation, Funding acquisition, Conceptualization. **Luis Ríos:** Writing – original draft, Investigation, Formal analysis, Data curation. **Patricio Arce-Johnson:** Validation, Methodology, Investigation. **Luisbel González:** Writing – review & editing, Software, Resources, Methodology, Funding acquisition, Formal analysis, Data curation, Conceptualization. **María Cristina Ravanal:** Writing – review & editing, Validation, Methodology, Investigation.

## Funding

This research was funded by FONDECYT-Chile, Project No. 1220263.

## Declaration of competing interest

The authors declare that they have no known competing financial interests or personal relationships that could have appeared to influence the work reported in this paper.

## Data Availability

Data will be made available on request.
